# Subjective Symptoms of First-Time Wearers of Verofilcon A Silicone Hydrogel One-Day Disposable Contact Lenses: A First Time Use of Verofilcon A (FTVeA) Study

**DOI:** 10.7759/cureus.62344

**Published:** 2024-06-13

**Authors:** Tatsuya Mimura, Masao Yamaguchi, Hidetaka Noma, Koichiro Shinbo

**Affiliations:** 1 Ophthalmology, Teikyo University School of Medicine, Tokyo, JPN; 2 Ophthalmology, Nerima Station West Eye Clinic, Tokyo, JPN; 3 Ophthalmology, Hachioji Medical Center, Tokyo Medical University, Hachioji, JPN

**Keywords:** questionnaire survey, verofilcon a, silicone hydrogel, glasses, first-time wearers, daily disposable soft contact lens

## Abstract

Purpose*:* Verofilcon A, a new daily disposable silicone hydrogel soft contact lens (SCL), is expected to be more comfortable to wear due to its smooth surface. This study aims to compare the comfort level of verofilcon A with eyeglasses in first-time SCL wearers.

Methods*: *A total of 58 new SCL wearers participated in this study. Participants' comfort scores while wearing glasses or verofilcon A were examined at baseline (glasses) and one and four weeks after starting to wear the SCLs.

Results*:* At the end of the one-month study period, no participants had abandoned wearing the SCLs due to discomfort. The vision scores (1-10) for glasses (baseline) and SCLs (week one, week four) were better with SCLs than glasses in the morning (7.9 ± 1.9 vs 8.9 ± 1.3, 9.0 ± 1.2, *p*<0.01), during the day (8.0 ± 1.6 vs 9.0 ± 1.1, 8.9 ± 1.2, *p*<0.01), at the end of the day (7.2 ± 2.1 vs 8.5 ± 1.5, 8.7 ± 1.4, *p*<0.01), and the entire day (7.7 ± 1.7 vs 8.9 ± 1.2, 8.7 ± 1.3,* p*<0.01). The percentages of participants who agreed that wearing SCLs at week four was as good as or better than glasses in terms of overall vision, peripheral vision, vision at the end of the day, comfort during the day, comfort at the end of the day, less fatigue during the day, and less fatigue at the end of the day were 91.4%, 100.0%, 91.4%, 89.7%, 82.8%, 87.9%, and 89.7%, respectively. Of the participants, 93.1%, 100.0%, and 93.1% felt that the SCLs were easy to wear, more comfortable than expected, and would like to purchase the same lenses in the future, respectively.

Conclusion*:* These findings suggest that verofilcon A is more comfortable than glasses and is effective as an introductory lens for first-time SCL wearers.

## Introduction

Contact lenses (CLs) are synthetic lenses worn on the surface of the eye to correct vision problems such as hyperopia, myopia, presbyopia, and astigmatism. The reasons for wearing CLs include the desire to wear them during sports or to live a more fashionable life free of glasses. Wearing CLs represents a major change in a person’s lifestyle. CLs have advantages over eyeglasses, including a wider field of vision and more natural vision; the frames of eyeglasses do not get in the way, they are visually unobtrusive, and they do not fog up or get wet in cold or rain like eyeglasses.

However, first-time CL wearers may give up on the first wear due to ocular irritation or may not continue wearing CLs because of dry eyes. In fact, a previous report examining the rate of CL wearers abandoning CL use found a dropout rate of approximately 40% of CL wearers within four months [1]. In addition, other reports have indicated that 50-75% of CL wearers experience discomfort with CLs and that 12-51% of wearers stop using CLs [1,2]. CLs used by first-time users should be comfortable and trouble-free for the wearer to promote their continued use.

Recently, silicone hydrogel (SiHy)-based soft CLs (SiHy SCLs) have appeared as one-day disposable soft CLs (SCLs). SiHy SCLs combine a conventional hydrogel material with a silicone material with high gas permeability and transparency for clinical applications [3]. SiHy SCLs have high gas permeability due to the silicone polymer, which provides sufficient oxygen permeability even at low water content, with less dryness, staining, and hyperemia [3]. By ensuring high oxygen permeability, SiHy allows us to reduce eye strain compared with SCLs made of conventional PMMA or HEMA materials. The evaluation of SiHy SCL comfort may provide important information for SCL users as the market for this product is expected to grow in the future.

Approximately eight types of SiHy SCLs are commercially available in Japan. In March 2021, a new daily disposable SCL made of silicon hydrogel called verofilcon A (PRECISION 1®, Alcon Japan Ltd. Tokyo, Japan) was launched. Verofilcon A is expected to be more comfortable to wear due to surface processing using SMARTSURFACE® technology and is expected to be particularly useful for first-time SCL wearers.

Verofilcon A was first launched in Oceania and the United States, and studies on its comfort have been conducted in Australia, New Zealand, and the United States [4,5]. A survey of 129 new CL wearers over the age of 18 in Australia and New Zealand found that 91% agreed that "verofilcon A is the preferred lens," 79% agreed that "verofilcon A offers an alternative to using glasses," and 70% agreed that "verofilcon A is the best lens to start the SCL wearing experience" [4]. In the United States, a crossover study was conducted on two types of SCLs, verofilcon A and etafilcon A, with 96 experiencing CL wearers [5], which found that 73.9% of participants preferred verofilcon A to etafilcon A. In addition, the ratings of visual acuity, SCL handling, and wearing comfort were significantly better for verofilcon A than for etafilcon A [5]. Thus, in North America and Oceania, verofilcon A has been reported to be easier to handle and provides better vision than conventional SCLs. However, as yet, no evaluations of the use of verofilcon A have been conducted in Asia, and a study on their wearing comfort is required.

This study aimed to compare the comfort of glasses and verofilcon A SCLs in first-time CL wearers and investigate their satisfaction with and tolerability of SCLs.

This article was previously posted to the medRxiv preprint server on April 27, 2023.

## Materials and methods

Research design

This was an investigator-initiated, observational, prospective cohort study. This study was conducted in accordance with the ethical guidelines of the Declaration of Helsinki (World Medical Association 2013) and the Ethical Guidelines for Medical Health. The studies involving human participants were approved by the Teikyo University Ethical Review Committee (#19-211, #20-166). A series of studies, including this one, have been registered as clinical trials in the University Medical Information Network for Clinical Trials (UMIN-CTR) (UMIN registration numbers: UMIN000041107 and UMIN000041107). This study was conducted between April 2021 and May 2022 at the outpatient clinic of the Nerima Station West Eye Clinic and the Department of Ophthalmology, Teikyo University School of Medicine. Written informed consent was obtained from all participants after a complete explanation of the study content.

Participants

The inclusion criteria were as follows: (1) healthy participants; (2) aged 12 years and older; (3) used glasses; (4) no history of CL wear; (5) myopic astigmatism; (6) refraction of -0.5D to -6.0D; and (7) best corrected visual acuity of 20/25 or better. The exclusion criteria were as follows: (1) aged < 12 years; (2) ocular or systemic disease; (3) history of refractive surgery; and (4) corneal epithelial erosion. Artificial tear drops were allowed for the CLs during the study. Participants were selected from patients who wanted to wear CLs for the first time. Therefore, a total of 58 participants were included. The age of the 58 patients ranged from 12 to 63 (mean ± deviation, 18.1 ± 9.3) years. A total of 22 males and 36 females participated in this study.

Study schedule

Objective refraction and the corneal radius of curvature were determined using an auto keratorefractometer (TONOREF® III Plus, TOPCON, Yamagata, Japan). The spherical power, cylindrical power, corneal radius, and minimum and maximum corneal refractive powers were measured. A spherical equivalent (sphere plus 1/2 cylinder) was used to calculate the refractive error. The corneal tear film break-up time (BUT) was measured with fluorescein under a slit-lamp or a tear interferometry DR-1α (Kowa, Nagoya, Japan). The BUT of both eyes was measured, and the average value was calculated.

The participants were asked to complete questionnaires about wearing eyeglasses and SCLs prior to the first SCL wear, one week after the first SCL wear, and four weeks after the first SCL wear. The registered participants underwent lens fitting by a CL specialist with more than 10 years of practical experience. Participants also received one hour of training and practice by a CL practitioner to insert and remove SCLs by themselves. The participants were instructed to wear the SCLs after they were able to wear them properly. They were instructed to wear the SCLs for 8-10 h per day. They were also asked to wear their SCLs five days a week and glasses on other days of the week. The subjects were instructed that the SCLs were intended for daily disposable use and were to be discarded after each day of wear. Examinations were conducted at baseline and after one and four weeks. A total of 58 participants were enrolled, and all 58 participants completed the one-month follow-up study.

Characteristics of the evaluated SCLs

Verofilcon A (PRECISION 1) daily disposable lenses made of SiHy were used. Verofilcon A is made from a new high oxygen permeability (Dk; 90 × 10−11 barriers) material with a 2-3 µm thick surface, more than 80% water content, and is a Class 1 ultraviolet blocker (≥ 90% of UVA, ≥ 99% of UVB) [6]. Verofilcon A SCLs have a smooth surface due to the SMARTSURFACE® technology.

Questionnaire

The questionnaire consisted of four parts: 1) a questionnaire on the comfort level of glasses at baseline, 2) a questionnaire on the quality of vision (QOV) of glasses or SCLs, 3) a questionnaire comparing glasses and SCLs, and 4) a questionnaire on the comfort and impression of wearing SCLs. We used the modified questionnaire scores used by Grant et al. and Marx et al. [4,7].

The first questionnaire consisted of the following four questions regarding comfort levels when wearing glasses at baseline: Q1: Eyes are comfortable overall with glasses; Q2. Peripheral vision was excellent with glasses; Q3: Eyes were comfortable throughout the day with glasses; Q4. The eyes were less tired throughout the day when wearing glasses. For each question, a five-point score was given: 1= strongly disagree, 2= disagree, 3= neither agree nor disagree (neutral), 4= agree, or 5= strongly agree.

The second questionnaire comprised four items related to the comfort and QOV of glasses and SCLs. As shown in Table 1, participants scored poor (1) to excellent (10) as to whether they were comfortable and whether their vision was good while wearing glasses or SCLs at insertion (morning), during the day (daytime), at the end of the day (evening), and overall (throughout the day) [4,7]. The questionnaires were administered before wearing the SCLs (at the baseline visit) and at one and four weeks after wearing the SCLs.

**Table 1:** Quality of vision (QOV) score (questionnaire 2). Modified scores used by Grant et al. and Marx et al. [4,7]. SCL: Soft contact lens

How good is your vision while using glasses or SCLs, on a scale of 1 to 10?										
	Poor Excellent									
Q1. At insertion (morning)	1	2	3	4	5	6	7	8	9	10
Q2. During the day (daytime)	1	2	3	4	5	6	7	8	9	10
Q3. At the end of the day (evening)	1	2	3	4	5	6	7	8	9	10
Q4. Overall (throughout the day)	1	2	3	4	5	6	7	8	9	10

The third questionnaire consisted of seven questions in which participants were asked whether they preferred glasses or SCLs in terms of vision and comfort (Table 2). Participants reported whether they preferred glasses or SCL on a scale of 1-5 (1= much better with glasses, 2= slightly better with glasses, 3= no difference between either, 4= slightly better with SCLs, and 5= much better with SCLs). The questionnaire was administered four weeks after the first SCLs were fitted.

**Table 2:** Questionnaire on whether eyeglasses or SCL are preferred (questionnaire 3). Modified scores used by Grant et al. and Marx et al. [4,7].

Questions about glasses vs. soft contact lenses (SCL). Please circle any 1-5.					
	Much better with glasses	Slightly better with glasses	No difference	Slightly better with SCLs	Much better with SCLs
Q1. Overall vision	1	2	3	4	5
Q2. Peripheral vision	1	2	3	4	5
Q3. Vision at the end of the day	1	2	3	4	5
Q4. Eyes are comfortable overall (during the day)	1	2	3	4	5
Q5. Eyes are comfortable at the end of the day (evening)	1	2	3	4	5
Q6. Eyes are less tired throughout the day	1	2	3	4	5
Q7. Eyes are less tired at the end of the day (evening)	1	2	3	4	5

The fourth questionnaire consisted of 13 items related to impressions of wearing SCLs, as shown in Table 3. The participants were asked whether they agreed (yes) or disagreed (no) with each item, and the questionnaire was administered four weeks after the SCLs were fitted.

**Table 3:** Questionnaire on SCL wear (questionnaire 4). Modified scores used by Grant et al. and Marx et al. [4,7]. SCL: Soft contact lens

How was your experience with contact lenses? Please circle (yes/no)		
Question	No	Yes
Q1. Easy to insert	0	1
Q2. Easy to wear	0	1
Q3. Convenient to use	0	1
Q4. Easier than expected to get used to	0	1
Q5. More comfortable than expected	0	1
Q6. As comfortable as no lenses at all	0	1
Q7. End of day as comfortable as beginning	0	1
Q8. So comfortable I don't feel anything	0	1
Q9. So comfortable I barely felt anything	0	1
Q10. So comfortable I forgot I was wearing them	0	1
Q11. Perfect when I choose not to wear glasses	0	1
Q12. I will recommend them to my friends	0	1
Q13. I am interested in purchasing them	0	1

Based on the scores of each item in the questionnaire, the severity of ocular symptoms and satisfaction with SCL comfort and vision were analyzed.

Statistical analyses

Two-tailed paired Student’s t-tests were used to determine the significance of differences between the two groups. A one-way analysis of variance and Bonferroni multiple comparison tests were performed to determine the measurements that were significantly different among the three groups. Correlations among the variables were determined by calculating two-tailed Pearson correlation coefficients. Data are expressed as the means ± standard deviation or percentage. Statistical analyses were performed using SAS System software version 9.1 (SAS Institute Inc., Cary, NC, USA), and significance was accepted at p <0.05.

## Results

Subject characteristics

The demographic and baseline characteristics of the participants are shown in Table 4. A total of 58 participants were enrolled. All 58 participants completed the study, and none dropped out. The right and left eyes of subjects had a similar mean refractive error (-3.9 ± 1.5 diopters (D) vs. -3.7 ± 1.4 D), mean sphere (-3.4 ± 1.5 D vs. -3.4 ± 1.4 D), and mean cylinder (-0.7 ± 0.4 D vs. -0.7 ± 0.4 D). The refractive power of the SCLs used by the participants was lower than that of their eyeglasses in both the right (-2.9 ± 1.5 D vs. -3.5 ± 1.5 D, p<0.01) and left eyes (-2.9 ± 1.5 D vs. -3.5 ± 1.5 D, p<0.01).

**Table 4:** Demographics and baseline characteristics of the participants. *refraction (D)=sphere+cylinder/2

Demographics/ baseline characteristics	
Age (years)	
Mean ± SD	18.1 ± 9.3
Range: minimum, maximum	12 to 63
Sex, n (%)	
Females	36 (62.1%)
Males	22 (37.9%)
Baseline refraction, mean ± SD, diopters (D)	
*Refractive error, right eye	-3.9 ± 1.5
*Refraction error, left eye	-3.7 ± 1.4
Sphere, right eye	-3.4 ± 1.5
Sphere, left eye	-3.4 ± 1.4
Cylinder, right eye	-0.7 ± 0.4
Cylinder, left eye	-0.7 ± 0.4
*Glasses refraction, right eye	-3.5 ± 1.5
*Glasses refraction, right eye	-3.5 ± 1.5
Contact lens power, right eye	-2.9 ± 1.5
Contact lens power, left eye	-2.9 ± 1.5
Baseline keratometry, mean (mm)	
Right eye K1 / right eye K2	7.9/7.7
Left eye K1 / left eye K2	7.9/7.7

Comfort level with glasses at baseline (questionnaire 1)

**Figure 1:**
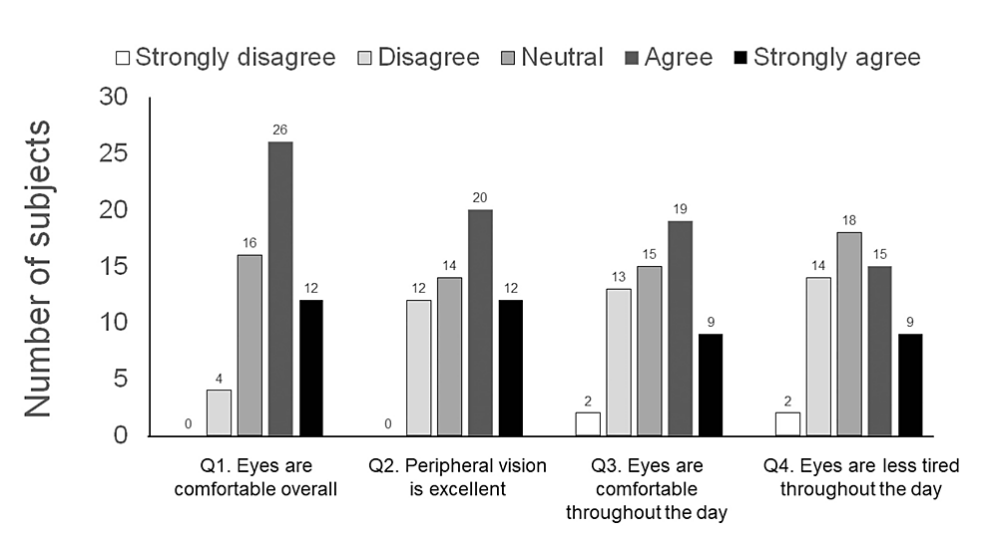
The ocular comfort level of participants wearing eyeglasses at baseline (n=58) (questionnaire 1).

The comfort levels of the participants wearing eyeglasses are shown in Figure 1. Furthermore, 38 of the 58 participants (65.5%) agreed or strongly agreed with ‘Q1: Eyes were comfortable overall with glasses’. Moreover, 32 of the 58 participants (55.2%) agreed or strongly agreed with ‘Q2: Peripheral vision is excellent with glasses’, 28 (48.3%) agreed or strongly agreed with ‘Q3: Eyes are comfortable throughout the day with glasses’, and 24 (41.4%) agreed or strongly agreed with ‘Q4: Eyes are less tired throughout the day’.

QOV score (questionnaire 2)

**Figure 2:**
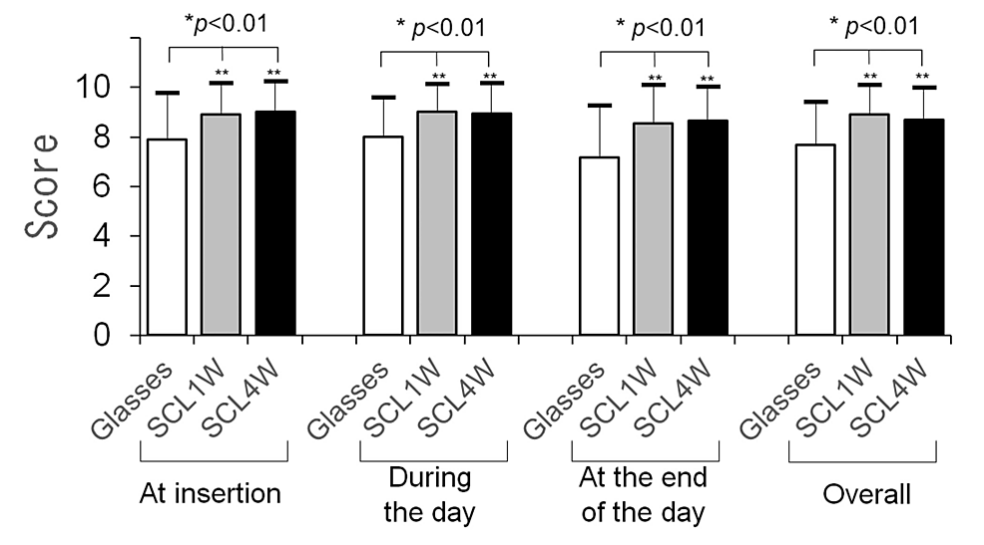
The quality of vision (QOV) scores with glasses at the baseline visit and soft contact lenses (SCL) at the one and four-week visits (n=58) (questionnaire 2). The results are reported on a scale of 1 (poor) to 10 (excellent). The results were compared among the three groups by a *one-way analysis of variance (p<0.01) and the **Bonferroni multiple comparison test (p<0.01, vs. habitual glasses).

The QOV scores (1-10) between glasses (baseline) and SCLs (week one, week four) were recorded in the morning (7.9 ± 1.9 vs. 8.9 ± 1.3 vs. 9.0 ± 1.2, respectively, p<0.01, Bonferroni multiple comparison test), during the day (8.0 ± 1.6 vs. 9.0 ± 1.1 vs. 8.9 ± 1.2, p<0.01), during desorption (7.2 ± 2.1 vs. 8.5 ± 1.5 vs. 8.7 ± 1.4, p<0.01), and overall (7.7 ± 1.7 vs. 8.9 ± 1.2 vs. 8.7 ± 1.3, p<0.01). SCLs had significantly higher QOV scores than eyeglasses (Figure 2).

Questionnaire on whether eyeglasses or SCL are preferred (questionnaire 3)

**Figure 3:**
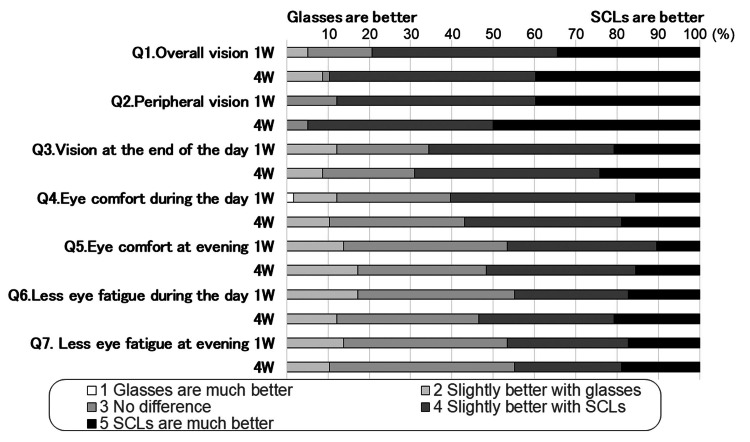
Evaluation of the quality of vision, comfort, and less eye fatigue between eyeglasses and SCLs (questionnaire 3). The participants scored whether they felt the eyeglasses or SCLs were preferable in response to the seven questions. For more details, refer to Table 3. SCL: Soft contact lens

The scale evaluation results of whether the glasses or SCLs were better are shown in Figure 3. The total percentages of scale 3 (no difference), 4 (slightly better with SCLs), and 5 (much better with SCLs) were calculated. The percentages of the total scores of 3, 4, and 5 for weeks one and four were as follows: Q1. Overall vision (94.8% at week one and 91.4% at week four), Q2. Peripheral vision (100.0% and 100.0%), Q3. Vision at the end of the day (87.9% and 91.4%), Q4. Eyes were comfortable overall (87.9% and 89.7%), Q5. Eyes were comfortable at the end of the day (86.2% and 82.8%), Q6. Eyes were less tired throughout the day (82.8% and 87.9%), and Q7. Eyes were less tired at the end of the day (86.2% and 89.7%). For all answers, the percentage of participants who thought that SCLs were better than glasses was higher.

Questionnaire on SCL wear (questionnaire 4)

**Figure 4:**
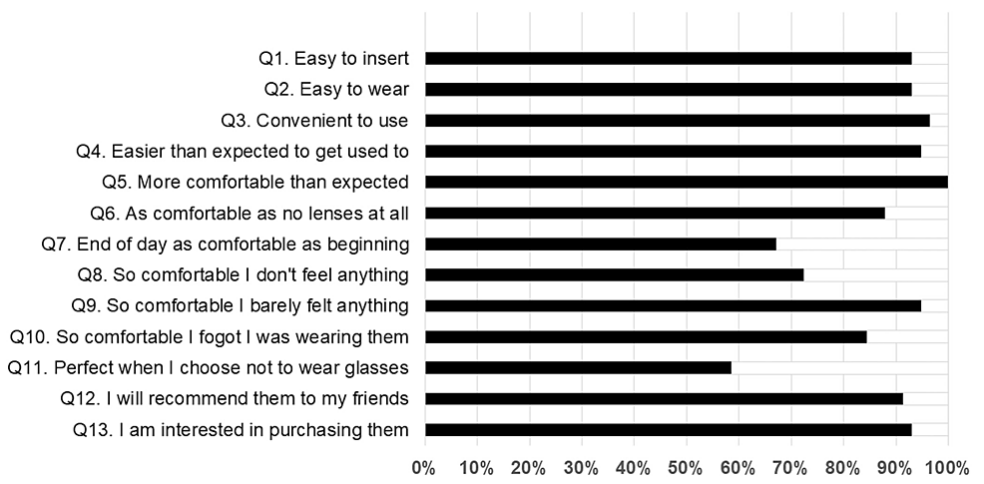
Evaluation or the wearer's impression and comfort with the SCL. The results are reported by participants regarding convenience, comfort, satisfaction, and purchase intentions for SCLs at four weeks. The participants answered “yes (agree)” or “no (disagree)” to the 13 questions (Questionnaire 4), as shown in Table 4. SCL: Soft contact lens

The questionnaire results regarding the wearers’ impressions of the SCLs and comfort are shown in Figure 4. The percentages of those who answered yes were as follows: Q1. Easy to insert, 93.1%; Q2. Easy to wear, 93.1%; Q3. Convenient to use, 96.6%; Q4. Easier than expected to become used to, 94.8%; Q5. More comfortable than expected, 100.0; Q6. As comfortable with no lenses at all, 87.9%, Q7. End of the day, as comfortable as the beginning, 67.2%; Q8. So comfortable, I don't feel anything, 72.4%; Q9. So comfortable, I barely felt anything, 94.8%; Q10. So comfortable, I forget I was wearing them, 84.5%; Q11. Perfect when I choose not to wear glasses, 58.6%; Q12. I will recommend them to my friends, 91.4%; and Q13. I am interested in purchasing them, 93.1%.

Relationship between the BUT and QOV score

**Figure 5:**
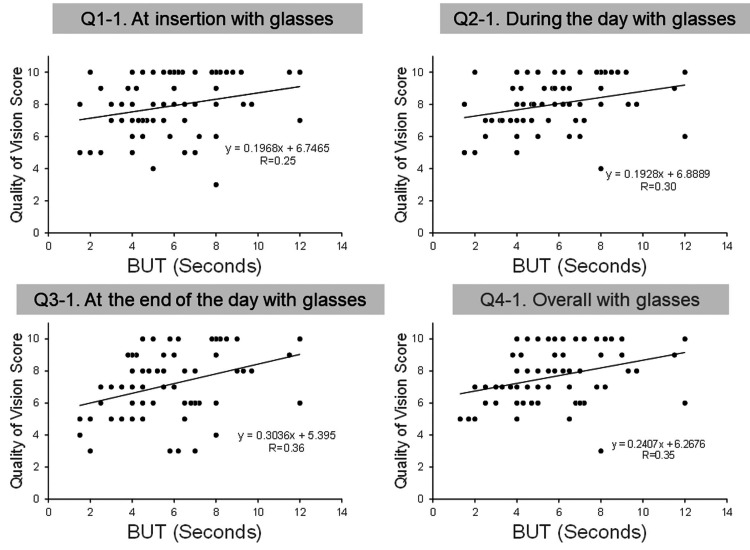
Relationship between the tear film break-up time (BUT) and the quality of vision (QOV) scores of glasses.

**Figure 6:**
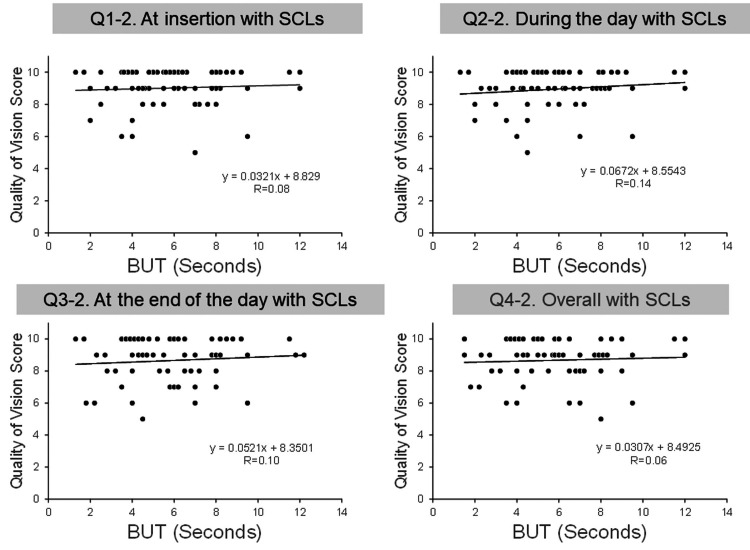
Relationship between the BUT and the SCL QOV score. BUT: Break-up time; SCL: soft contact lens; QOV: quality of vision

The mean BUT of both eyes at baseline was 5.9 ± 2.5 s (range 1.5-12.0 s). The correlations between the BUT and QOV scores for glasses and SCLs are shown in Table 5, and their scatter plots are shown in Figures 5, 6. All four glasses QOV scores correlated with the BUT (Figure 5, all p<0.05), but none of the SCL QOV scores correlated with the BUT (Figure 6).

**Table 5:** Relationship between the tear film break-up time (BUT) and QOV score of glasses or SCLs. Correlations between the tear film break-up time (BUT) and quality of vision (QOV) scores of glasses or SCLs were calculated using Pearson’s product-moment formula. R: Pearson’s correlation coefficient; CI: confidence interval; SCL: soft contact lens

		Correlation Coefficients			
Variables	R		(95% CI)	p-value	
Glasses					
Q1-1. At insertion with glasses	0.25		(-0.01-0.48)	0.03	
Q2-1. During the day with glasses	0.30		(0.05-0.52)	0.01	
Q3-1. At the end of the day with glasses	0.36		(0.11-0.57)	<0.01	
Q4-1. Overall with glasses	0.35		(0.10-0.56)	<0.01	
SCL					
Q1-2. At insertion with SCLs	0.08		(-0.19-0.33)	0.29	
Q2-2. During the day with SCLs	0.14		-0.13-0.38)	0.16	
Q3-2. At the end of the day with SCLs	0.10		-0.17-0.35	0.24	
Q4-2. Overall with SCLs	0.06		-0.20-0.31)	0.33	

## Discussion

Summary of the results

Our results showed that all QOV scores were higher with SCLs than with glasses in the morning, during the day, when they were removed, and at any time during the day. The wearers also had a better impression of SCLs than glasses in terms of vision, comfort, and eyestrain. In the questionnaire on impressions of wearing SCLs, participants generally had a good impression (58.6-100%) of all 13 items. The BUT affected the participants' vision with glasses but not with CLs. This suggests that verofilcon A provides comfortable vision, even with dry eyes. In addition, these results suggest that verofilcon A is more comfortable than glasses and is effective as an introductory lens for first-time CL wearers.

Comfort and discontinuation of SCLs

In the present study, almost all 58 patients could comfortably use the SCLs for one month without any problems. There were no adverse events, and none of the patients complained of any problems in relation to wearing the SCLs. The discomfort experienced when wearing SCLs can lead to their discontinuation. In a previously reported study of 4207 patients, 40% abandoned wearing SCLs within four months [8]. Compared to patients who discontinued wearing SCLs, those who did not wore more SiHy SCLs (49% vs. 38%) [8]. The paper reported that the main reasons for abandoning SCL use were discomfort (24%), followed by dryness (20%), hyperemia (7%), and cost (7%) [8]. In another study, 110 subjects (ages 13 to 19) who had never worn CLs before were randomized to wear either nelfilcon A SCLs (DAILIES® AQUACOMFORT PLUS®, Alcon) or glasses for six months [9]. By the sixth month of the study, 13 of the 110 patients had discontinued from the study. Of these, 10 (17.5%) were in the SCL group, and three (5.7%) were in the glasses group. The percentage of subjects who discontinued treatment was significantly higher in the SCL group than in the glasses group (p=0.04) [9]. Although the duration of our study was only one month, which is shorter than that of the other studies mentioned, no patient discontinued wearing the SCLs during the study period. One reason for this may be that the comfort provided by verofilcon A was comparable to or better than other SCLs. Another reason is that ophthalmologists and practitioners provided a detailed explanation of the SCL-fitting process to the participants prior to the start of the fitting, which may have contributed to the reduction in dropouts.

Comparison of QOV between SCL and glasses

With respect to questionnaire 2, the participants indicated that their QOV with SCLs was clear at all examination times (morning, daytime, and night), even when compared with glasses. The SCLs were fitted with a lower power compared to the power of the glasses used by the participants, but the corrected visual acuity of the two was similar. In a study that included a total of 110 teenagers and compared a group of first-time SCL wearers to a group of glasses wearers, visual acuity was reported to be better in both groups at all study visits [9]. In contrast, facial appearance (p<0.001), satisfaction with SCL wear (p<0.001), daily activity (p<0.001), peer recognition (p=0.003), and the overall score (p<0.001) were higher in the first-time SCL group than the glasses group [9]. These results, including ours, indicate that the QOV is better with SCLs than with glasses and that quality of life is more comfortable with SCLs than with glasses.

Another survey on wearing verofilcon A SCLs was conducted in New Zealand and Australia [4]. This study recruited 218 patients who had already used one-day disposable SCLs and had switched from other SCLs to verofilcon A, a SiHy SCL, and 129 first-time CL wearers who were using verofilcon A. Of the 129 first-time CL wearers, 75% of them agreed that the SCLs were comfortable to wear throughout the day, 78% agreed that the SCLs provided as clear a view at the end of the day as at the beginning of the day, and 79% agreed that wearing SCLs provided clear vision of text and pictures on digital devices [4]. This study and our results show that verofilcon A can provide clear vision for first-time SCL users.

Comparison of comfort SCL and eyeglasses

The results of questionnaire 3 showed that the percentage of participants who reported that the SCLs were as good as or better than glasses at week four of the study were as follows: vision (91.4% overall and 91.4% at the end of the day), eye comfort (89.7% overall and 82.8% at the end of the day), and less eye fatigue (87.9% overall and 89.7% at the end of the day). These results indicate that approximately 90% of participants agreed that wearing SCLs was at least as comfortable as wearing eyeglasses. Why is verofilcon A far more efficacious than glasses in terms of vision, comfort, and eye strain? SCL discomfort and dry eyes are the most significant factors that make SCLs unsatisfactory to wear [10-12]. In a report of 4207 cases, approximately 23% of the study participants eventually stopped wearing CLs permanently, and the main reasons for dropout were persistent discomfort and dryness [8]. The dropout rate has been reported to be lower for wearers with SiHy SCLs [8]. In other words, a better way to reduce SCL dropout would be to make SCLs more comfortable to wear and alleviate dry eye symptoms. On the other hand, approximately 10% of participants preferred glasses to SCL (Figure 3). Therefore, it is important to keep in mind that while wearing SCLs can improve the QOV and quality of life, some people are more comfortable with glasses.

Innovations in SiHy SCLs

SiHy combines two materials: a hydrophobic silicone with excellent oxygen permeability and a hydrophilic polymer. It has high oxygen permeability and generally low water content [13]. On the other hand, verofilcon A is a lens with extremely high oxygen permeability (Dk: 90) and oxygen transmissibility (Dk/L: 100), which are characteristics of SiHy, and also overcome the disadvantage of SiHy, which is low water content (high water content; center of SCL: 51% water content; near SCL surface: 80% or more) [4]. These special features of verofilcon A may increase the adhesion and moisture retention of the SCL to the ocular surface and enhance the satisfaction of SCL wearers.

Various materials are available for SiHy SCLs, some of which have poor water retention properties and are uncomfortable to wear. To overcome the shortcomings of these SiHy materials, verofilcon A has been shown to improve water retention and comfort. This proprietary technology is called "SMARTSURFACE® technology" and has been used in the manufacturing process of verofilcon A [4]. Verofilcon A achieves a very high water content of over 80% by covering the SCL surface with a hydrophilic polymer that retains water. The key manufacturing process for SMARTSURFACE® technology is the immersion of SCL in a liquid filled with hydrogel polymer and a water-soluble polymer containing polyacrylic acid (PAA). PAA is a highly hydrophilic polymer that absorbs large amounts of water to form a hydrogel. The highly hydrophilic gel-like PAA gives verofilcon a special structure with 80% water content on the SCL surface and 51% water content in the core of the SCL [4]. According to the manufacturer's instructions, during the processing stage of SMARTSURFACE® technology, this hydrophilic polymer solution expands the lens material, creating small, narrow pores 2-3 microns in diameter on the SCL surface [4]. The hydrophilic PAA polymer penetrates the open pores and is anchored within the hole, forming an ultrathin moisture layer. Additionally, the heating process crosslinks the PAA polymer with a wetting agent consisting of a copolymer of polyamidoamine-epichlorohydrin and polyacrylamide-acrylic acid (PAAm-PAA) [4]. A thin layer covered with PAA on the lens surface continuously stores moisture and helps stabilize the tear film on the ocular surface. The moisture stored on the surface of the SCL provides smoothness to the verofilcon A surface. According to the company literature, these characteristics lead to long-lasting comfort and a clear vision. The moderately rigid structure of the central portion of the SCL and its high moisture content makes it easier to fit and remove [4]. Our study demonstrated that approximately 90% of first-time SCL wearers were able to easily fit the SCL (Figure 4). In addition, Grant et al. also reported that 82% of first-time SCL wearers agreed that the verofilcon A SCLs were easy to wear, and 72% agreed that verofilcon A SCLs could be easily removed at the end of the day [4]. In addition, verofilcon A [14,15] and delefilcon A [16] SCLs have been shown by other surveys to provide high-quality vision, a comfortable fit, and easy handling [14-16].

Surface characteristics of verofilcon A SCLs

SMARTSURFACE® technology not only improves the fit of the SCL but also reduces the adhesion of protein and air dust to the SCL surface. The results of our recent study comparing 12 different SCLs showed that verofilcon A SCLs had lower pollen attachment than other SCLs [17]. In general, SCLs made of HEMA have a higher protein adhesion than SiHy SCLs [18]. In addition, SiHy SCLs are coated with a hydrogel-rich material to improve the wettability of the SCL surface [18]. This unique SCL surface processing structure not only reduces friction between the ocular surface and the inside of the SCLs but also protects against the adhesion of proteins and foreign particles to the SCL surface. Our previous studies have demonstrated that verofilcon A has very low adhesion to atmospheric particles, mainly pollen and Asian dust components, compared to lenses made of other SiHy materials [17,19]. These results suggest that the low friction of the SCL and the extremely high oxygen permeability and water content of verofilcon A lead to patient satisfaction.

High satisfaction and ease of use among first-time wearers of SCLs

The results of questionnaire 4 regarding their impressions of wearing verofilcon A SCLs were as follows: 93.1%, 93.1%, 96.6%, 94.8%, and 100.0% could easily place the SCL in their eyes, could easily wear the SCL, could easily use the SCL, found the SCL easier to use than expected, and found the SCL more comfortable than expected, respectively. In a survey of 129 first-time verofilcon A wearers, 91% agreed that "verofilcon A gives me the option of not using glasses," 79% agreed that "it is the best lens to start my contact lens wearing experience," and 70% agreed that "verofilcon A is the SCL I would like to use in the future” [4]. These study results suggest that the processing of the verofilcon A surface by SMARTSURFACE® technology made a favorable impression on first-time SCL wearers. In the current study, first-time SCL wearers were highly satisfied with using verofilcon A. In fact, 93.1% of participants agreed that they would purchase these lenses in the future (Figure 4). High satisfaction with the use of SCLs may also be attributed to the practitioners and ophthalmologists providing detailed instructions on proper SCL wear and the advantages and disadvantages of SCLs.

Study limitations

This study had several limitations. First, this was a one-arm observational study, not a randomized, two-group comparative study. A one-arm before-and-after study may have resulted in subjective bias. Second, the study duration was one month, and future long-term studies on verofilcon A wear are needed. Third, most study participants were young because they were first-time contact lens wearers. Future studies should be conducted with a wider range of age groups. Fourth, this study was primarily based on a questionnaire survey of the participants. Additional investigations of other findings, such as tear fluid dynamics and lens movement during verofilcon A wear, would be useful.

## Conclusions

In conclusion, the comfort and QOV of verofilcon A SCLs were better than those of eyeglasses for first-time SCL wearers. None of the participants stopped using the SCLs because of problems, and most agreed that verofilcon A SCLs were so comfortable that they did not notice they were wearing CLs. Verofilcon A may be useful as an introductory lens for first-time SCL users.
